# The mushroom matrix: an engineered mycelium-derived biochar platform for advanced biotechnological applications

**DOI:** 10.3389/fbioe.2025.1707953

**Published:** 2025-11-24

**Authors:** Xingguo Tian, Tian-Ye Du, Wenhua Lu, Jaturong Kumla, Entaj Tarafder, Tikka Dewage Chamarika Priyadarshani, Rekhani Hansika Perera, Kalani Kanchana Hapuarachchi, Nakarin Suwannarach

**Affiliations:** 1 School of Food and Pharmaceutical Engineering, Guizhou Institute of Technology, Guiyang, Guizhou, China; 2 Guizhou Key Laboratory of Agricultural Microbiology, Guizhou Academy of Agricultural Sciences, Guiyang, China; 3 Yunnan International Joint Laboratory for Digital Conservation and Germplasm Innovation and Application of China-Laos Tea Resources, School of Tea and Coffee, Pu’er University, Pu’er, China; 4 Center of Excellence in Microbial Diversity and Sustainable Utilization, Chiang Mai University, Chiang Mai, Thailand; 5 Department of Biology, Faculty of Science, Chiang Mai University, Chiang Mai, Thailand; 6 Center for Yunnan Plateau Biological Resources Protection and Utilization and Yunnan International Joint Laboratory of Fungal Sustainable Utilization in South and Southeast Asia, College of Biology and Food Engineering, Qujing Normal University, Qujing, China; 7 Department of Plant Science, Faculty of Agriculture, Rajarata University of Sri Lanka, Anuradhapura, Sri Lanka; 8 Zest Lanka International (Private) Limited, Polonnaruwa, Sri Lanka; 9 College of Biodiversity Conservation, Southwest Forestry University, Kunming, China

**Keywords:** bioremediation, biostimulation, microbial carrier, microbiome, mycomaterials, pyrolysis, sustainable biotechnology

## Abstract

Mushroom cultivation generates vast amounts of spent substrate, while the controlled growth of fungal mycelium offers a dedicated feedstock for advanced materials. This review synthesizes the science of mushroom-derived biochars, a distinct class of biomaterials sourced from this underutilized biomass. This review demonstrates that the inherent biological architecture of fungal matter, specifically its chitinous framework and nitrogen-rich composition, is preserved through pyrolysis to create biochars with superior functionality. We demonstrate how these materials transcend their traditional role as soil amendments to serve as programmable platforms for biotechnology. The review explores how pyrolysis parameters and advanced synthesis methods, such as chemical activation and co-pyrolysis, can be precisely tuned to engineer bespoke properties, including ultra-high surface areas (>1200 m^2^/g) and enhanced contaminant affinity. A central focus is placed on the unique capacity of mushroom biochars to act as prebiotic scaffolds that directly modulate microbial communities, drive biogeochemical cycles, and facilitate breakthrough applications. By mapping the journey from mycelium to advanced mycomaterials, this work charts a course for the intentional design of tailored myco-materials to address pressing global challenges in environmental remediation, sustainable agriculture, energy storage, and sensing technologies.

## 1 Introduction

Humans have been utilizing fungi for food and medicine for thousands of years, yet now we are on the brink of harnessing their complex biology for the creation of next-generation materials. At the forefront is the mushroom, not only as a food product, but as an innovation model (Manan et al., 2021; Zou G. et al., 2024). The global cultivation sector generates millions of tonnes annually of spent mushroom substrate (SMS), a lignocellulosic waste stream that also represents a significant environmental issue (Guo et al., 2022). Concurrently, controlled fermentation of fungal mycelium provides the chance to produce dedicated, high-purity biomass. This nexus of feedstocks and wastes creates a new paradigm: the transformation of fungal biomass into designed biochars, beyond waste valorization and toward the frontier of precise biological design (Gu et al., 2023; Zhang B. et al., 2024; Zhang L. et al., 2024; Zhang X. et al., 2024).

In this review, we employ a tiered and precise terminology to categorize these fungus-derived biochars, which is crucial for understanding their properties and applications. The general term “mushroom-derived biochar” serves as the overarching category for all carbonaceous materials produced via pyrolysis of fungal biomass. This broad category is divided into two primary, chemically distinct feedstocks. The first is “Spent Mushroom Substrate biochar (SMS-biochar)”, derived from the solid waste left over after cultivating fruiting bodies. This is a composite material composed of a partially degraded lignocellulosic substrate and the embedded mycelium of a cultivated mushroom species. Its properties are highly dependent on both the fungal species and the original substrate composition, a key variable that will be specified for cited studies. The second is “Mycelial biochar” (or “Myco-char”), reserved for biochar produced from purpose-grown, pure mycelial biomass. This feedstock offers a more uniform, nitrogen-rich, and tunable chemical composition, primarily derived from the fungal cell wall. While “mushroom-derived biochar” is used as the general term, the specific designations “SMS-biochar” and “Mycelial biochar” will be used rigorously throughout to highlight the critical distinctions rooted in feedstock origin and resultant material properties.

**FIGURE 1:**
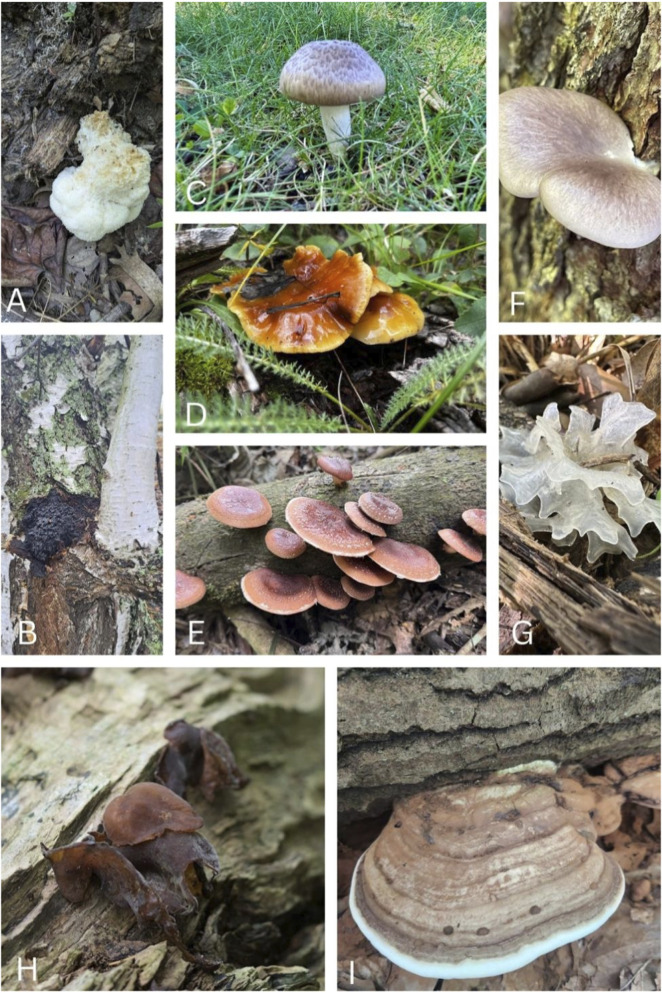
Representative mushroom species whose mycelium or spent cultivation substrates are used in biochar production. **(A)** *Hericium erinaceus* (Russulales). **(B)** *Inonotus obliquus* (Hymenochaetales). **(C)** *Agaricus bisporus* (Agaricales). **(D)** *Flammulina velutipes* (Agaricales). **(E)** *Lentinula edodes* (Agaricales). **(F)** *Pleurotus ostreatus* (Agaricales). **(G)** *Tremella fuciformis* (Tremellales). **(H)** *Auricularia auricula*-*judae* (Auriculariales). **(I)** *Ganoderma applanatum* (Polyporales). (https://www.inaturalist.org/, the images are used under the license Attribution Non-Commercial- No Derivs 4.0.

Biochar is a carbonaceous material typically produced from lignocellulosic biomass, i.e., wood or crop waste, through pyrolysis in a state of restricted oxygen (Akdeniz, 2019; Varkolu et al., 2025). Because of its porous structure and chemical characteristics, biochar can play an important role in enhancing agronomic soils and overall environmental welfare (Zhu et al., 2025). In addition, other wastes of mushroom spent substrate and fungi are a cheap and renewable feedstock for the manufacture of biochar, which is an auspicious technique of agricultural waste recycling founded on principles of circular bioeconomy (Aiduang et al., 2025). Several mushrooms employed in biochar production are illustrated in Figure 1.

Mycelium and mushroom biochars represent an elite and specialized group of biomaterials. Model functionality of the materials owes directly to the intrinsic biological ancestry of the materials (Chen et al., 2022). The chitinous structure of the fungal cell wall, an important nitrogenous polymer that is not prevalent in plant feedstocks, is preserved through pyrolysis to create a surface with a high-density population of reactive functional groups (Zhu L. et al., 2021; Crognale et al., 2022). This, along with hierarchical porosity that echoes the intricate architecture of the hyphal network, results in a material with record-breaking cation exchange capacity (CEC), surface reactivity, and innate biocompatibility (Domingues et al., 2020; Wijitkosum et al., 2025). It is this unique composition that transforms these types of biochars from passive amendments to active prebiotic platforms with the capability to modulate microbial populations, enhance soil fertility, and sequester environmental pollutants with great efficiency (Dai et al., 2021; Zhang H. et al., 2022; Zhang M. et al., 2022; Kumar et al., 2025).

Whereas previous reviews have accurately documented the agronomic use of SMS-based biochar, this article argues for a paradigmatic expansion of scope. This paper introduces the concept of the “mushroom matrix,” a multifunctional biological scaffold from which numerous classes of functional biochars can be developed. We critically synthesise the feedstock-to-function pathway, describing how the biological source (SMS or pure mycelium) dictates final material properties and how pyrolysis conditions can be engineered to achieve designer properties for target applications. Our scope encompasses the fundamental science of biochar-microbe interactions, as well as their nascent biotechnological applications, including microbial carriers for bioaugmentation, designed ecosystems for bioremediation, and beyond. By mapping out this wide design space, this review aims to establish a foundation context for the intentional development of myco-materials, positioning mushroom-based biochar as a keystone technology in addressing pressing global challenges in environmental sustainability and green technology. Furthermore, in this review, we introduce the concept of “mycomaterials”, an umbrella term for functional materials derived from fungal biomass, including spent substrate, pure mycelium, and processed fungal polymers, which exhibit tailored properties for advanced biotechnological applications.

## 2 The feedstock spectrum

The nature of the end biochar is a direct consequence of its biological source. Appreciation of this spectrum from waste to carefully considered biomass is the first step toward conscious design (Wang and Wang, 2019). SMS is a complex blend of decomposed lignocellulose, mycelial, and metabolic residues. This complex nature results in an SMS-biochar with high mineral content, moderate porosity, and significantly higher nitrogen content compared to plant-derived biochars. While typical plant residue biochars (e.g., from wood or straw) are characteristically nitrogen-poor (<0.5%), SMS biochar exhibits a significantly enriched nitrogen content (0.44%–2.71%) (Fu et al., 2024; Aiduang et al., 2025). This nitrogen enrichment is further amplified in biochar from purpose-grown, pure mycelial biomass (“myco-char”), which can achieve nitrogen levels of 5%–10%, owing to the high chitin content of the fungal cell wall. This elevated nitrogen content, a direct consequence of residual fungal chitin, along with a hierarchical pore structure, makes fungal-derived biochar a robust workhorse material, extremely effective in large-scale soil amendment and environmental remediation plans (Jin et al., 2021).

**FIGURE 2:**
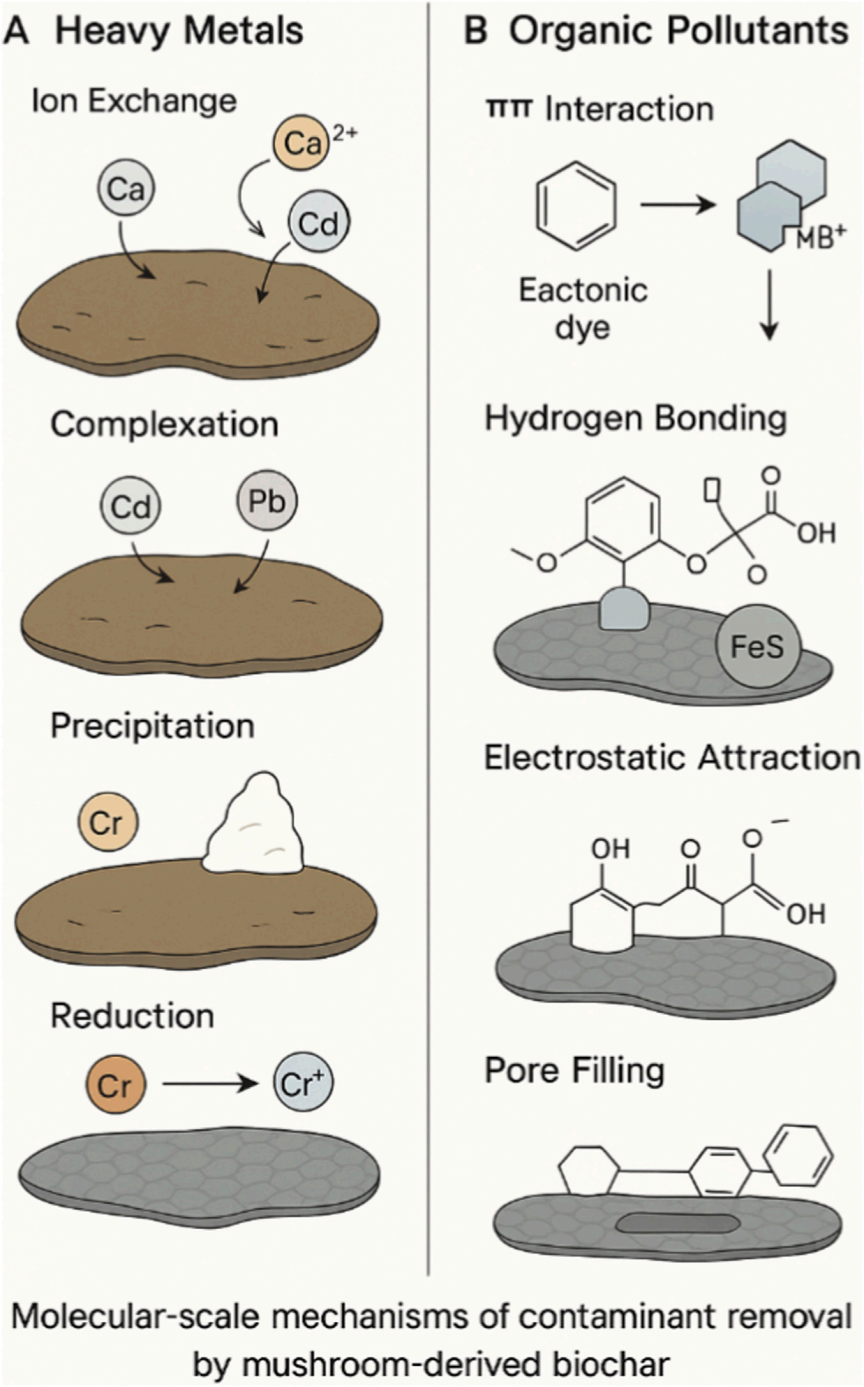
Mechanisms of contaminant removal by mushroom biochar. Illustrations of the principal molecular-scale mechanisms for adsorption of **(A)** heavy metals and **(B)** organic contaminants. The primary mechanisms for metals are ion exchange, surface complexation, precipitation, and reduction; for organics, these include π–π interactions, electrostatic attraction, hydrogen bonding, and pore filling.

Its inherent porosity, which directly solidifies the hyphal network, is highly effective in retaining water and nutrients in a growing substrate, providing adequate sites for microbial colonization and contaminant adsorption (Aiduang et al., 2025). Figure 2 represents the primary mechanisms by which mushroom-derived biochar removes contaminants, highlighting ion exchange, surface complexation, precipitation, and reduction for heavy metals, as well as π–π interactions, electrostatic attraction, hydrogen bonding, and pore filling for organic pollutants.

Purpose-grown, pure mycelium (for instance, *Aspergillus* or *Rhizopus* species) use is a significant step towards engineered biochars. The process has a number of benefits (Gu et al., 2023; Zhang B. et al., 2024; Zhang L. et al., 2024; Zhang X. et al., 2024). Pure chitin composition results in high and uniform nitrogen enrichment, resulting in high pyrolysis density of reactive functional groups like pyridinic-N and pyrrolic-N. Prior to pyrolysis, physical mycelium architecture can be regulated. For instance, liquid fermentation yields pellets, while solid-state fermentation yields complex networks, directly influencing pore size distribution and surface area of the biochar (Kasera et al., 2022). Lam et al. (2019) demonstrated the effect of physical architecture by preparing SMS-biochar from *P. ostreatus* substrate via microwave vacuum pyrolysis, with a surface area of 215 m^2^/g. On the other hand, those derived from pure, engineered mycelial networks achieve significantly higher scores, even exceeding 1200 m^2^/g after activation, revealing the immense potential of this tailor-made feedstock (Crognale et al., 2022). The most sophisticated approach is using purified fungal materials, such as chitin and chitosan, as feedstocks. This targeted tool strategy produces biochars with very specific and homogenous surface chemistries, and these are most useful for high value uses where uniformity is important, such as in biomedicine or precision catalysis (Azuma et al., 2014; Bąk et al., 2022).

In a large-scale comparison of 29 waste-derived biochars, biochar from spent mushroom substrate of *Auricularia auricula* (AAMB) ranked among the best-performing adsorbents. It possessed a unique structure of closely compacted micron-sized channels, a high surface area (341.1 m^2^/g), and an enormous total pore volume (0.149 cm^3^/g). Its superior physicochemical properties enabled strongly effective adsorption of methylene blue by monolayer chemical adsorption and intra-particle diffusion. The study highlights the critical importance of feedstock selection in ascertaining biochar performance and firmly identifies spent mushroom substrate as a good precursor for developing effective adsorbents (Han et al., 2023). To fully contextualize the unique value of fungal-derived feedstocks, it is critical to directly contrast their properties with those of conventional plant-derived biochars. The distinct biological origins of these materials result in fundamental differences in their biochar characteristics, as summarized in Table 1. These differential properties dictate the specific scenarios and applications where mushroom-derived biochars offer superior or unique functionality.

**TABLE 1:** Comparative analysis of key characteristics between mushroom-derived biochars and conventional plant-derived biochars.

Feature	Mushroom-derived biochar (SMS and myco-char)	Typical plant-derived biochar (e.g., wood, straw)	Critical implication
Nitrogen content	High (0.44%–10%) due to chitin and proteins	Low (<0.5–1.5%)	Superior for nitrogen-involving catalysis, as a microbial nutrient source, and for N-doped carbon materials
Inherent porosity	Hierarchical, preserving hyphal network	Varies; often requires activation to achieve high surface area	Provides a natural, high-surface-area scaffold ideal for microbial colonization and contaminant adsorption
Surface functionality	N-rich groups (pyridinic-N, pyrrolic-N) from chitin	O-rich groups (carboxyl, hydroxyl) from lignin/cellulose	Different contaminant affinity. Fungal biochars may be better for complexing certain metals and organics via different mechanisms
Cation exchange capacity (CEC)	Inherently higher due to N-functional groups	Generally lower, more dependent on pyrolysis temperature	Better nutrient retention in agricultural applications
Ash and mineral content	Can be high and variable in SMS-biochar	Typically lower, more consistent in woody biochars	Can be a pro (source of minerals) or con (may reduce carbon content and stability). This is a key point for critical discussion
Feedstock uniformity	Myco-char offers high tunability; SMS is a composite	Woody biochar is relatively consistent; agricultural waste is variable	Myco-char is superior for engineered, high-value applications where consistency is key

## 3 Pyrolysis as a design tool

Pyrolysis is the pivotal process that transforms biological feedstocks into valued biochar, and its parameters are powerful levers for designing end properties (Leng and Huang, 2018; Tomczyk et al., 2020). Pyrolysis temperature is the primary regulator of the character of biochar. Low-Temperature Pyrolysis (300 °C–500 °C) maximizes the preservation of labile, nitrogen- and oxygen-rich functional groups from fungal chitin and proteins (Janu et al., 2021; De Oliveira et al., 2024). These groups play an essential role in cation exchange and nutrient retention, and hence low temperature biochars are ideal for land use in agriculture where fertility augmentation and nutrient cycling are the aims (Rasse et al., 2022; Kabir et al., 2023). The carbon structure is not as stable and this might limit long-term sequestration. High-Temperature Pyrolysis (>600 °C) triggers extensive aromatization and the formation of stable graphitic carbon structures. It leads to a substantial increase in surface area, microporosity, and heat stability (Zhang et al., 2020; Tu et al., 2022; Li L. et al., 2023).

The pyrolysis surface functional group manipulation as a strategic process is also shown in a systematic study of three SMS-biochars from *Hypsizygus marmoreus*, *Pleurotus geesteranus*, and *Lentinula edodes* (Li et al., 2019). FTIR analysis indicated that high temperatures and prolonged pyrolysis times consistently promoted aromatization (broadening of C-C groups) while degrading labile oxygen- and nitrogen-containing surface functional groups (C=O, C-O-C, C-N); the most stable structure was obtained at 700 °C for 3.0 h. Significantly, the study also indicated the intrinsic impact of the biological feedstock, such that at the same pyrolysis conditions, *Hypsizygus marmoreus* biochar retained the highest oxygen functional groups, hinting at an excellent ability for heavy metal or organic pollutant absorption, while the highest aromatic structure was yielded by *Pleurotus geesteranus* biochar, which reflects the best ability for carbon sequestration. This study emphasizes the dual principles of design in choosing both the best feedstock and precise pyrolysis conditions to design the functionality of biochar (Li et al., 2019).

As demonstrated by Chen et al. (2022), the pore property of biochar of *Auricularia auricula-judae* (black fungus) and *Lentinula edodes* substrate showed a significant enhancement with the increase in the temperature of pyrolysis from 400 °C to 600 °C, whereas the biochars synthesized at 800 °C showed large BET surface areas of 312.5 and 280.9 m^2^/g. While certain functional groups are decomposed, research indicates that ample oxygen-containing functional groups remain, suggesting inherent stability in SMS feedstocks. These materials are superior to contaminant sorption and sequestering carbon in the long term. Carbon sequestration potential may be significantly enhanced by planned modification; for instance, iron-doped mushroom biochar exhibited higher carbon holding (12.2%–44.5%) and heat stability, and 60.6% sequestration efficiency at 600 °C because iron catalyzed graphitization and inhibited volatile organic compound emission. This demonstrates how targeted chemical modification can enhance specific functional properties like carbon sequestration efficiency (Liu et al., 2024a). An exhaustive report on biochar from *Tremella fuciformis, Flammulina velutipes,* and *L. edodes* substrates confirmed the worldwide trends: biochar yield and content of volatile matter decrease at elevated pyrolysis temperatures, and pH and ash increase (Zhao et al., 2019).

Besides conventional pyrolysis, new technologies can additionally improve biochar characteristics. Chemical Activation involves the pre-treatment of the feedstock with a chemical agent before pyrolysis that dramatically alters the outcome. For instance, phosphoric acid (H_3_PO_4_) activation can produce activated biochars with extremely high surface areas (>1200 m^2^/g) and extremely mesoporous character (>82%), which is appropriate for specific adsorption applications (Grimm et al., 2024). Co-Pyrolysis, thermal conversion of mixed feedstock, can potentially utilize synergistic properties. Innovative research by Sewu et al. (2017) demonstrated that co-pyrolysis between SMS and ashy seaweed (*Saccharina japonica*) resulted in a biochar with an adsorption capacity 2.2 times greater for dye than pure SMS-biochar due to the ash component in seaweed being responsible for increasing the functional groups and surface properties of the biochar.

## 4 The biological interface

**FIGURE 3:**
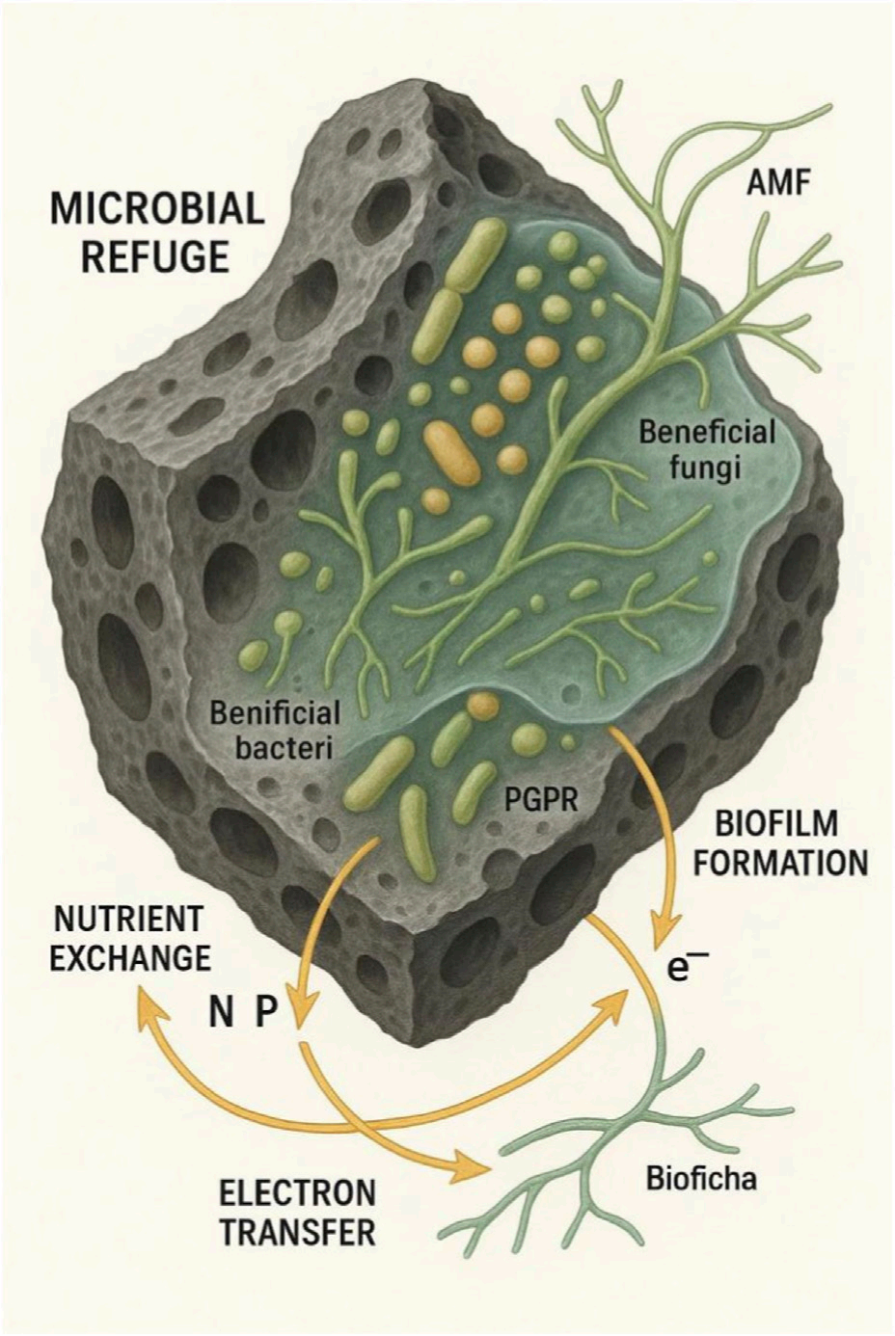
The Biochar-Microbe interface.

The true potential of mushroom biochars is the way they engage with biological processes in a dynamic manner as more than inert sorbents (Li et al., 2020; Kracmarova-Farren et al., 2024; Zhang L. et al., 2024). The chitin-derived microfibers and the nitrogen-functional groups are the perfect source of nutrients and substrate for colonization by Plant Growth-Promoting Rhizobacteria (PGPR) and Arbuscular Mycorrhizal Fungi (AMF), the soil beneficial microbes (Jabborova et al., 2021; Guan et al., 2023; Figure 3).

This specific inoculation is a stimulator of microbial diversity and activity, which is absolutely important for nutrient cycling, decomposition of organic matter, and equilibrium within ecosystems. The porosity of the matrix provides microenvironmental niches that shield these microbes from predation and environmental distress, in effect a refuge for a healthy microbiome (Ren et al., 2022; Zhang et al., 2023; Zou Q. et al., 2024). The habitat of biofilm created on biochar possesses the ability to regulate microbial sociality. It can suppress quorum sensing in pathogenic bacteria, rendering them less virulent, and simultaneously facilitate syntrophic interaction in consortia of pollutant-degrading microbes (Mukherjee et al., 2022; Cheng et al., 2024; Li et al., 2024). This manipulation of microbial ecology is among the most crucial mechanisms of action for its bioremediation potential. For example, Kong et al. (2022) showed in a research study that the addition of SMS to compost enhanced Cd, Cr, and Pb passivation by 25.47%–47.91%. This action was attributed to the biochar’s role in promoting microbial metabolism, primarily by providing a protective habitat and facilitating electron transfer processes that enhanced microbial-mediated metal stabilization and humification during the composting process.

## 5 Mycomaterials for advanced biotechnological applications

The unique features of mushroom-derived biochars, nitrogen-rich functionality, hierarchical porosity, and surface chemistry that is controllable—spur their application much beyond conventional soil conditioning. This section discusses their advanced applications in three main areas: environmental remediation, agricultural biotechnology, and novel materials technology, demonstrating the groundbreaking potential of the “mushroom matrix.”

### 5.1 Sophisticated environmental remediation

Mushroom biochars are superior engineered materials for water and soil remediation, leveraging their excellent surface reactivity and binding capacity for inorganic and organic pollutants.

#### 5.1.1 Remediation of heavy metals and metalloids

Mushroom-derived biochars are highly effective for heavy metal and metalloid remediation from water and terrestrial environments, coupling waste valorization with environmental cleanup. Adsorption efficiency is feedstock, pyrolysis temperature, and chemical or nano-modification dependent.

##### 5.1.1.1 Feedstock and pyrolysis effects

SMS-biochar from *Agaricus bisporus* (compost-based substrate) has temperature-dependent processes: ion exchange (∼350 °C), π-electron coordination (∼550 °C), and mineral precipitation (∼750 °C), with maximum Cu adsorption of 68.1 mg·g^−1^ (Zhang et al., 2021). Waste mushroom-stick biochars (300 °C–800 °C) have a trade-off between oxygen-containing groups being maintained in low-temperature biochars and high-temperature biochars having increased alkalinity, ash content, aromaticity, and polarity, which enhance adsorption of Pb^2+^, Cu^2+^, Cd^2+^, and Ni^2+^ (Wang X. et al., 2019). Biochars of *Flammulina velutipes, Pleurotus ostreatus, Auricularia auricula*, and *Lentinula edodes* (350 °C–600 °C) indicate that high-lignin substrates favor surface complexation or mineral precipitation, while others include precipitation and Cu^2+^–π interactions, with Cu (II) adsorption changing 52.6–65.6 mg·g^−1^ (Jin et al., 2021). Biochars derived from mushrooms are chemically more homogeneous compared to lignocellulosic feedstocks, therefore being more uniform in adsorption performance (Liu Z. et al., 2022).

##### 5.1.1.2 Nano- and chemical modifications

Sulfur-modified *Pleurotus ostreatus* biochar upgraded Cd (II) adsorption to 55.96 mg·g^−1^, 229% higher than that of unmodified biochar, via sulfur complexation, precipitation, and Cd–Cπ interaction (Liu M. et al., 2022). Nano-Fe_3_O_4_- and nano-FeS-coated *Lentinula edodes* biochars extracted Cr (VI) with efficiencies of 99.44% and 99.57 mg·g^−1^, respectively, converting Cr (VI) to Cr (III) with long-term stability (Wang C. et al., 2019; Wang J. et al., 2024). Fe/Mn-modified *Hericium erinaceus* biochar removed Cd (II) and phosphate (100% and 95%) by surface complexation and precipitation (Dickson and Cornish, 2024). Hydroxyapatite–biochar composites and iron-doped biochars achieved Pb adsorption uptakes of up to 243.07 mg·g^−1^ through dissolution–precipitation, ion exchange, cation–π interactions, and surface complexation (Lee et al., 2024; Fan et al., 2025).

##### 5.1.1.3 Applications in soil and plant responses

SMS biochar enhances soil fertility and reduces metal bioavailability. Maize that was subjected to Cd and Cr improved height (26.1%), root dry weight (99.7%), grain yield (98.2%), and chlorophyll content (50%) (Dawar et al., 2025). Alfalfa (*Medicago sativa*) grown in Cd-, As-, and Cu-polluted soil experienced a 44% reduction in Cd-available in the soil, higher biomass, enhanced enzyme activity, and richer microbial communities (Wang et al., 2023). *Pleurotus djamor* SMS-biochar reduced arsenic levels in rice grains by up to 27% and increased yield by 14.6%–18.5% and micronutrient value, an exemplar of circular economy valorization (Mridha and Roychowdhury, 2024). Soil amendments with SMS-biochar also mitigated cadmium contamination under severe nitrogen stress, reducing bioavailable Cd by 28%–29.5%, increasing soil pH, cation exchange capacity, organic matter, and stimulating microbial activity, including recovery of ammonia-oxidizing bacteria (Li et al., 2022). Biochar aerogel composites, such as hydroxyapatite-modified mushroom biochar immobilized within calcium alginate beads, exhibited good Pb^2+^ (564.5 mg·g^−1^) and Cd^2+^ (302.2 mg·g^-1^) adsorption with recovery and reusability as scalable remediation strategies (Ji et al., 2023).

##### 5.1.1.4 Mechanisms and modeling

**FIGURE 4:**
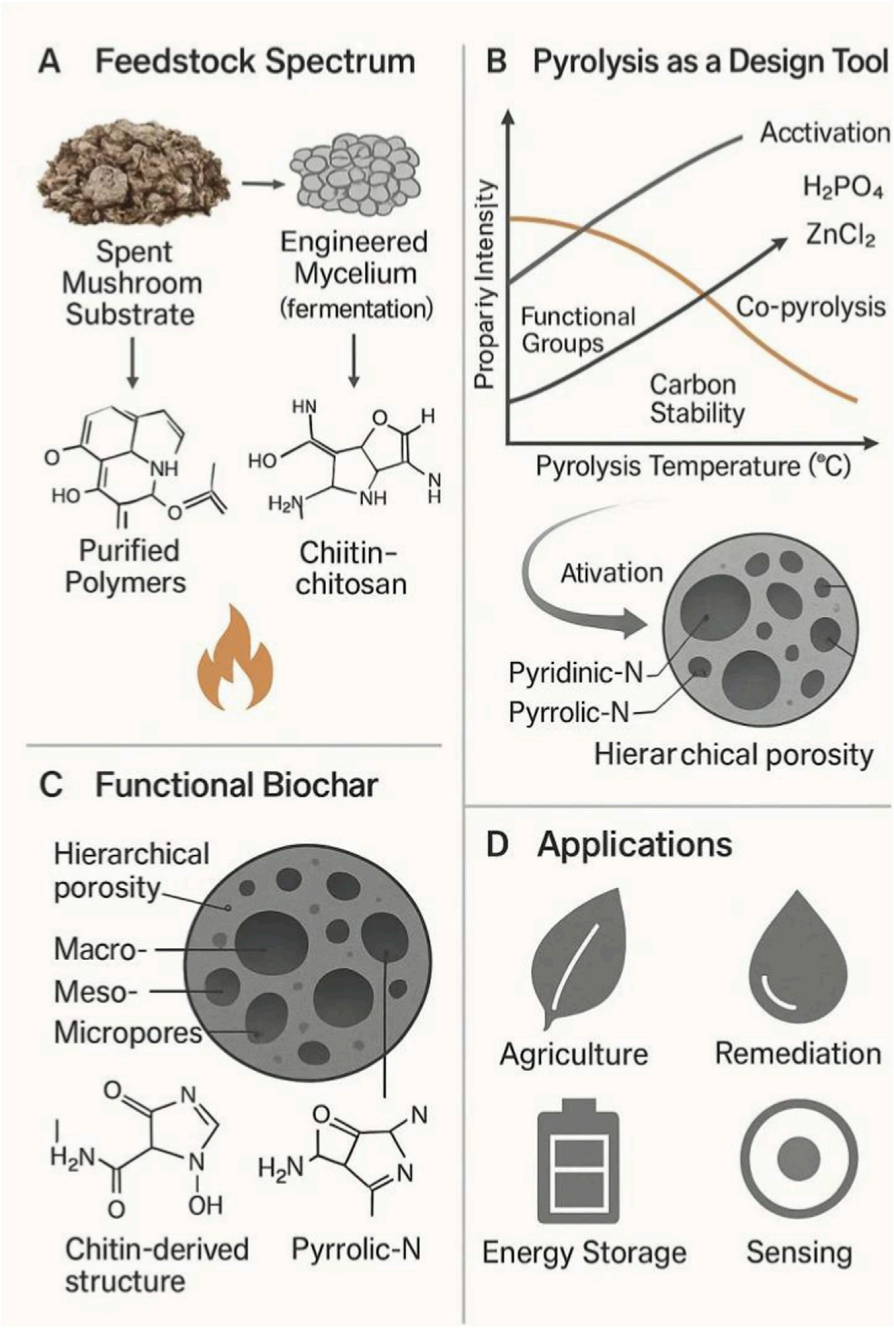
Key molecular mechanisms of action for environmental remediation using mushroom biochar. **(A)** Feedstock spectrum. **(B)** Pyrolysis as a design tool. **(C)** Functional biochar. **(D)**. Applications.

Adsorption processes are typically endothermic, involving intraparticle diffusion, and have Langmuir and pseudo-second-order mechanisms. Machine learning models, such as the Adaptive Neuro-Fuzzy Inference System (ANFIS), can effectively model adsorption efficiency for Pb, Cu, Fe, and Mn, enabling optimization in mining-affected waters (Abdallah et al., 2019; Madzin et al., 2024). Mushroom biochars, especially when chemically or nano-modified, offer multi-functional, high-performance heavy metal and metalloid remediation products based on mechanisms of ion exchange, surface complexation, precipitation, redox transformation, and π-interactions (Arabzadeh et al., 2024; Ahmed and Aidi, 2025; Madzin et al., 2025). Efficiency is established through feedstock selection, pyrolysis conditions, and modifications, with soil and agricultural applications demonstrating practical and sustainable applications (Sarfraz et al., 2019; Zhang X. et al., 2025). Figure 4 illustrates the key molecular mechanisms of mushroom biochar in environmental remediation, highlighting its chitin-derived nitrogen-functionalized surface and heterogeneous porosity that enable the removal of inorganic pollutants (e.g., Pb^2+^, Cd^2+^, CrO_4_^2-^) and organic pollutants (e.g., dyes, antibiotics, endocrine disruptors), thereby underscoring its multifunctional role in water and soil cleansing. Table 2 summarizes studies on the use of mushroom-derived biochar for the removal of various metal contaminants.

**TABLE 2:** Mushroom-derived biochar for metal contaminant removal.

Feedstock	Feedstock type	Substrate/Growth medium	Modification	Target contaminant	Max capacity (qmax, mg·g^-1^)	Experimental conditions (pH/temp./contact time)	Primary mechanism	References
*Agaricus bisporus* SMS	SMS-biochar	Compost-based substrate	None (temp. series)	Cu (II)	68.1	5.0/25 °C/24 h	Ion exchange, π-coordination, precipitation	Zhang et al. (2021)
*Auricularia auricula-judae*		Sawdust-based substrate (sticks)	None (temp. series)	Pb (II)	234.2	5.0/25 °C/24 h	Complexation, ion exchange, precipitation	Ji et al. (2022)
*Hericium erinaceus* waste		Spent substrate (composite)	Fe/Mn-modification	Cd (II) and phosphate	100% and 95% removal	6.0/25 °C/120 min (Cd2+) 4.0/25 °C/120 min (PO43-)	Surface complexation, precipitation (multi-functional)	Dickson and Cornish (2024)
*Lentinula edodes* SMS		Sawdust (80%) and bran (20%)	None	Pb (II)	398.0	5.0/25 °C/12 h	Precipitation, complexation	Wu et al. (2019)
		Wood chips and wheat bran	Nano-Fe_3_O_4_ coating	Cr (VI)	99.44% removal	2.0/30 °C/120 min	Adsorption, reduction to Cr (III)	Wang C. et al. (2019)
	Fruiting body biochar	N/A (direct biomass)	Nano-FeS modification	Cr (VI)	99.57	6.0/30 °C/24 h	Adsorption, chemical reduction	Wang Z. et al. (2024)
*Pleurotus ostreatus* SMS	SMS-biochar	Agricultural waste composite	Sulfur-modification	Cd (II)	55.96	6.0/25 °C/120 min	S-complexation, precipitation, Cd–π bonding	Liu M. et al. (2022)

#### 5.1.2 Organic pollutant removal (dyes, antibiotics, EDCs)

Besides heavy metals, white-rot fungi biochars from mushrooms also remove organic pollutants by sorption and catalytic degradation. In aquaculture, SMS-biochar improved pond conditions in red claw crayfish culture by increasing pH and reducing toxic ammonia-N and sulfide, thereby improving overall water quality (Lin et al., 2021). *Inonotus obliquus* residue (sclerotium)-derived Zn-modified high-performance biochar (Zn-IORBC) achieved 1676.78 m^2^/g surface area, 1.87 cm^3^/g pore volume, and adsorption efficiency of methylene blue (1033.66 mg·g^−1^) and tetracycline (947.42 mg·g^−1^) and long-term efficacy in natural aquatic systems (Shi et al., 2022). This surface area is highly competitive, often surpassing that of many commercial activated carbons, which typically range from 500 to 1500 m^2^/g. SMS-biochar pyrolyzed at 600 °C efficiently adsorbed endocrine-disrupting compounds, progesterone (232.64 mg·g^−1^) and 17α-ethinylestradiol (138.98 mg·g^−1^), with >80% removal in continuous-flow tests (Vieira et al., 2022). SMS-biochar from *L*. *edodes* bran (PBC300–PBC700) adsorbed tetracycline (7.57–17.68 mg·g^−1^), with surface characteristics and adsorption controlled by pyrolysis temperature and forecasted using machine learning (Liu M. et al., 2022). Magnetic SMS-biochar from *L. edodes* enabled separation of pyrethroid insecticides and enabled sensitive detection using HPLC (Zhao et al., 2024).

Integrated SMS-biochar–*Herbaspirillum huttiense* (SMSB-HHS1) composites simultaneously removed Cu, Zn, oxytetracycline, and enrofloxacin through chemisorption and biodegradation (Zhang X. et al., 2024). Nitrogen-doped SMS-biochar from *L. edodes* improved reactive orange-16 adsorption from 168 mg·g^−1^–393 mg·g^−1^ and demonstrated efficient removal from authentic effluents (Grimm et al., 2023). SMS-biochar from Djulis (*Chenopodium formosanum*) SMS biochar achieved 41.16 mg·g^−1^ methylene blue adsorption, while calcium-rich *G. lucidum* and *L. edodes* biochars immobilized Malachite Green (9,388.04 mg·g^−1^) and Safranine T (3,871.48 mg·g^−1^) via pore filling, electrostatic forces, and π–π stacking (Zhang M. et al., 2022; Morgan et al., 2025).

Scale-up SMS-biochar production and steam activation (SA-BC) increased BET surface area (332 m^2^/g) and adsorption of cationic dyes like crystal violet (1057 mg·g^−1^), reducing COD and color in real wastewater (Sewu et al., 2019). SMS-biochar from wheat straw pretreated by *Pleurotus pulmonarius* enhanced porosity for nitrate adsorption capacity of 0.37 mg·g^−1^, which was double that for untreated biochar (Wu et al., 2024). Hydrochar from *P. ostreatus* spent substrate, activated with K_2_CO_3_ or thiourea, adsorbed ciprofloxacin (>84% in 5 min), highlighting the potential of hydrothermal carbonization as a substitute to pyrolysis for antibiotic removal effectively (Romero et al., 2025). Magnetic biochars achieved methylene blue adsorption of 2297.04 μg/g with multi-cycle stability (Liu et al., 2024b).

Fungal-activated biochars effectively adsorbed herbicides, tetracycline, and Cr (VI) with spontaneous and exothermic interactions and multi-cycle reusability (Li Y. et al., 2023; Eltaweil et al., 2024; Achira et al., 2025). Magnetic hydrochars and MOF composites on mushroom biochar efficiently removed Sb (III) (636.9 mg·g^−1^), Sb(V) (814.3 mg·g^−1^), and Sb^3+^ (56.49 mg·g^−1^) via ion exchange, complexation, co-precipitation, and oxidation with perfect recyclability (Zhu G. et al., 2021; Duan et al., 2024). *Pleurotus ostreatus* magnetic biochar was also a long-lasting carrier of laccase and efficiently degraded bisphenol A, estradiol, and ethinylestradiol (>80–90% within 24 h) and served as an adsorbent and enzyme support during biocatalytic wastewater treatment (Yu et al., 2025). SMS-biochar improved microbial metabolic function, diversity, and hydrocarbon degradation in petroleum-contaminated soils during soil remediation and had the potential for waste recycling and ecosystem restoration (Hu W. et al., 2025). Phosphoric acid-activated biochar from *I*. *obliquus* residue (sclerotium) (P-IORBC) created ultra-high surface area (2014.51 m^2^/g) and high adsorption for tetracycline and cationic dyes, better than Zn-activated biochar, and highlights medicinal mushroom residues as renewable feedstocks for high-performance adsorbents (Guo et al., 2023).

Hence, mushroom biochars, including unipolar and chemically modified ones, represent multifunctional, high-performance devices for the clean-up of soil and water from heavy metals, dyes, antibiotics, endocrine disruptors, nutrients, and herbicides. Adsorption, chemisorption, ion exchange, π–π interactions, redox processes, precipitation, and catalytic degradation are at play, allowing both green waste valorization as well as environmental cleaning. Table 3 summarizes the properties of mushroom-derived biochars, their modifications, and their efficacy in removing organic pollutants from aqueous systems.

**TABLE 3:** Mushroom-derived biochars and their modifications for the removal of organic pollutants.

Biochar source	Modification/treatment	Target pollutant	Pollutant type	Max adsorption capacity (mg·g^-1^)	Experimental conditions	Mechanism	References
*Chenopodium formosanum* (Djulis) SMS	Pyrolyzed 800 °C	Methylene blue	Dye	41.16	7.0/25 °C/24 h	Physisorption, heterogeneous surface	Morgan et al. (2025)
*Ganoderma lucidum* + *Lentinus edodes* SMS	Calcium-rich	Malachite green, Safranine T	Dye	9,388.04, 3,871.48	Mg: 8.0/25 °C/24 h ST: 4.0/25 °C/24 h	Pore filling, electrostatic, π–π stacking	Zhang H. et al. (2022)
*Herbaspirillum huttiense*	Composite	OTC, ENR, Cu, Zn	Antibiotic/Metal	–	-	Chemisorption + biodegradation	Zhang X. et al. (2024)
*Inonotus obliquus*	Fe-loaded	Tetracycline	Antibiotic	183.81	-/25 °C/720 min	Adsorption + catalytic activity	Li Y. et al. (2023)
	Zn-modified	Methylene blue, tetracycline	Dye, antibiotic	1033.66, 947.42	-/25 °C/360 min	Electrostatic attraction, H-bonding, π–π interactions, pore filling	Shi et al. (2022)
*L. edodes* SMS	N-doped	Reactive orange-16	Dye	393	6.0 (natural)/22 °C/210 min (3.5 h)	N-functional groups, adsorption	Grimm et al. (2023)
	Magnetic	Pyrethroid pesticides	Organic pollutant	–	Unadjusted/25 °C/30 min	Surface functionalization, adsorption	Zhao et al. (2024)
*L. edodes* bran	300 °C–700 °C	Tetracycline	Antibiotic	7.57–17.68	6.0/25 °C/120 min	Chemical adsorption	Liu M. et al. (2022)
*Pleurotus ostreatus* SMS	Magnetic, laccase immobilized	BPA, E2, EE2	EDCs	80%–90% removal	5.0/30 °C/24 h	Enzyme-catalyzed degradation + adsorption	Yu et al. (2025)
SMS	Magnetic	Methylene blue	Dye	2297.04	Unadjusted/25 °C/30 min	Porous, magnetically separable	Liu et al. (2024b)
	Steam-activated	Crystal violet	Dye	1057	∼7.0 (Unadjusted)/25 °C/1440 min	Increased BET surface area, chemisorption	Sewu et al. (2019)
SMS pyrolyzed 600 °C	None	Progesterone, EE2	EDCs	232.64, 138.98	9.56/25 °C/1440 min	Adsorption via porous surface	Vieira et al. (2022)

#### 5.1.3 Catalysis and advanced oxidation processes (AOPs)

Although mushroom-derived biochar no longer contains active fungal enzymes, it can still catalyze the degradation of various contaminants through non-enzymatic pathways. The catalytic activity primarily stems from abundant surface functional groups (e.g., –OH, –COOH), redox-active metal oxides, and graphitic carbon domains that facilitate electron transfer and adsorption–oxidation reactions. These physicochemical features enable biochar to act as an efficient catalyst or catalyst support, even in the absence of biological activity (Gasim et al., 2022; Ye et al., 2025). Mushroom-derived biochars have been successfully utilized as catalysts to degrade organic pollutants in water via advanced oxidation processes. Biochar from *G. applanatum* residue (MBC-800) activated peroxymonosulfate (PMS) efficiently, achieving 88.83% removal of ketoprofen (KTP). Characterization revealed a sieve tube structure, a high surface area (632.08 m^2^/g), and a high content of functional groups, with ketone and carboxyl groups serving as active sites. Both nonradical and radical processes played a role, with sulfate radicals (SO_4_^−^) being the primary KTP oxidizers, recognizing mushroom biochar as an elevated-performance biomass-derived activator (Liu et al., 2025).

A biochar (Fe-BC) composite of zero-valent iron from edible mushroom residue dregs activated PMS for malachite green dye degradation, achieving 98% removal in 20 min within a wide pH range (5–9), with efficiency retained after five reuse cycles. Electron paramagnetic resonance (EPR) and quenching analyses identified sulfate radicals (SO_4_^−^), hydroxyl radicals (OH), and singlet oxygen (^1^O_2_) as predominant reactive species, verifying the stability and efficiency of functionalized mushroom biochars for AOPs (Wang et al., 2025).

Fe–N-doped SMS-biochar (Fe-N-BC) from spent mushroom substrate (SMS) at 900 °C also catalyzed persulfate degradation of tetracycline, achieving 95% removal in 120 min. High-temperature pyrolysis enhanced Fe oxide development and active sites, facilitating a synergy of adsorption, radical (mainly SO_4_^−^), and non-radical processes. The catalyst was 76% effective after five cycles, pointing towards the potential of high-temperature doped SMS-biochars as green, robust catalysts for antibiotic treatment (Xu et al., 2025).

#### 5.1.4 Nutrient recovery from wastewater

Mushroom-derived biochars exhibit higher efficiency for targeted nutrient recovery and purification of contaminated aqueous streams, transforming contaminants into valuable resources. One of the major applications is the recovery of excess phosphate and nitrate, which can cause eutrophication. Mg-Fe-modified SMS-biochar (Mg-Fe@BSMW) exhibited a high adsorption capacity for phosphate as 247 mg·g^−1^ with pseudo-second-order kinetics and Langmuir isotherm kinetics, which indicates spontaneous, exothermic monolayer chemisorption (Alhujaily et al., 2020). Similarly, iron-amended SMS-biochar (SMCB/Fe) adsorbed nitrate efficiently with a maximum adsorption capacity of 19.88 mg·g^−1^ at pH 5–7, and kinetics confirming chemisorption as the dominant process (Darajeh et al., 2021). SMS-biochar (SMSB) is equally suitable for ammonia-N capture and adsorbs 12.6 mg·g^−1^ by physical forces dominated by acidic functional groups (Halim et al., 2017).

Aside from adsorption, mushroom biochar also supports advanced nutrient recycling technologies. Used with biogas slurry, it facilitates nutrient recovery through struvite precipitation. When the process was conducted under optimal pH 9 conditions, the removal efficiencies reached 71% for ammonium (NH_4_^+^) and 99% for phosphate (P), with the recovered phosphate in the form of valuable struvite crystals. The efficiency of recovery was also enhanced by high pyrolysis temperatures and the addition of common ions (K^+^, Zn^2+^, Fe^3+^, CO_3_^2-^), solidifying its position as a recyclable product for circular nutrient management (Kubar et al., 2021).

The use of mushroom biochar ranges from decontaminating harmful anions and metalloids to producing clean drinking water. SMS-biochar (SMCB) with an aluminum coating effectively removed fluoride (36.5 mg·g^−1^) over a broad range of pH values (6–8) and has proven to be a suitable biosorbent for fluoride removal (Chen et al., 2016). Likewise, MnO_2_-modified SMS-biochar (from mushroom-cultivation “sticks”) exhibited a strong affinity for antimony and adsorbed Sb (III) at 50–64 mg·g^−1^ via chemisorption and partial oxidation to the less toxic Sb(V) (Mao et al., 2022).

### 5.2 Agricultural biotechnology

In agriculture, the role of mushroom biochar is evolving from a simple soil amendment to a multifunctional biotechnological tool for enhancing sustainability and closing resource loops.

#### 5.2.1 Soil fertility and crop productivity

Mushroom-derived biochar, particularly SMS-biochar, is a very effective fertilizer used for soil amendment to increase plant growth and soil fertility. It enhances the physicochemical properties of soils, such as SMS-biochar from *Pleurotus* ostreatus produced at 450 °C–600 °C, which enhanced organic matter in soil, total carbon and nitrogen, exchangeable potassium, cation exchange capacity, and porosity and reduced bulk density, leading to increased Chinese cabbage and Welsh onion growth (Jang et al., 2023). Similarly, SMS-biochar effectively enhanced the pH, nutrient supply (N, P, K), germination rate, shoot growth, tillering, and switchgrass biomass production in acidic soils (Xiang et al., 2025).

For heavy metal-contaminated soils, mushroom-derived biochar demonstrates remarkable remediation potential. Composite amendments combining spent mushroom substrate with its derived SMS-biochar reduced exchangeable cadmium by 28.3%–29.5% in contaminated soils even under high nitrogen levels, while simultaneously improving soil pH, organic matter content, and microbial biomass (Li et al., 2022). Similarly, SMS-biochar from *Pleurotus djamor*, when applied at 1%, reduced arsenic accumulation in rice grains by 27% while increasing grain yield by 18.5%, demonstrating the dual benefits of contaminant mitigation and productivity enhancement (Mridha and Roychowdhury, 2024). It also has implications for nutrient cycling and retention. Lou et al. (2017) demonstrated that SMS-biochar composite reduced leaching of Total Nitrogen (TN) by 43% and organic contaminant leaching (estimated as CODCr) by 66% compared to SMC. Additionally, the addition of SMS-biochar at 4% to calcareous soils significantly enhanced the growth of tomato, yield, photosynthetic pigments, nutrient content, fruit quality, and antioxidant activity, while also showing promise for improving crop productivity in nutrient-poor soils (Sardar et al., 2025). Under salinity stress, spent mushroom compost (SMC) and its derived SMS-biochar promoted parsley growth and nutrient uptake, and the greatest effect was offered by a 3% biochar addition in enhancing water retention and alleviating salinity effects (Karami et al., 2019).

Alongside chemical fertilizers, mushroom-derived biochar is a microbial inoculant carrier, transferring a porous and shielding matrix for beneficial fungi and bacteria. It enhances symbiotic associations as a mycorrhizal helper to ease colonization and nutrient exchange. Described as co-association with PGPR application, it continued to experience increased growth, yield, and biochemical processes in cauliflower, proving its two-fold role as an inoculant carrier and microbial benefactor (Širić et al., 2022).

Soil application is exceptionally beneficial to the environment through the reduction of greenhouse gas emissions. *Lentinula edodes* SMS-biochar influenced CO_2_ and N_2_O emission in moso bamboo forest soil, where 450 °C biochar had the tendency to reduce nitrogen processes in general, but high-temperature biochar compensated waste management and GHG regulation through the modification of dissolved C/N, pH, and microbial community structure (Deng et al., 2020). Similarly, SMS-biochar in *Camellia oleifera* soils reduced N_2_O emissions by 92.9% at 120% water holding capacity but increased CH_4_ under flooding, pointing to the fact that its climate benefit is feedstock- and moisture-sensitive (Xu et al., 2022).

#### 5.2.2 Composting and waste valorization

SMS-biochar significantly enhances the composting process and improves the utilization of organic waste. When incorporated into dairy or pig manure-based compost mixtures, SMS-biochar increases lignocellulose degradation, extends the thermophilic phase, enhances microbial diversity, and improves nitrogen retention, hence enhancing the final compost quality (Zhang et al., 2014; Zhang T. et al., 2025). The agronomic value of this SMS-biochar-amended compost is illustrated by the yield increases of up to 49% achieved through its application on rice (Zhang et al., 2014).

Soil field trials on degraded soils demonstrate that SMS-biochar, applied together with inorganic fertilizer, raises soil pH, organic carbon, total nitrogen, phosphorus, potassium, and the C/N ratio, but has little impact on physical properties, such as bulk density (Mao et al., 2018). Besides nutrient enhancement, SMS-biochar has also been proven to enhance the biological safety of compost. In a chicken manure experiment, 5% (dry weight) SMS-biochar effectively enhanced the removal of antibiotic-resistant genes (ARGs) and pathogenic bacteria. Notably, the poor performance of biochar from rice straw highlighted the superiority of SMS-biochar and underscored the importance of selecting feedstock carefully to mitigate biological risks in composting (Cui et al., 2016).

#### 5.2.3 Mycotechnology and cultivation improvements

Completing a circular economy loop, mushroom-derived biochar is applied back into mycotechnology to improve cultivation itself. A key challenge in cultivating high-value fungi like *G. lucidum* is slow mycelial growth, which can be mitigated by amending standard growth media (e.g., PDA and sorghum) with low concentrations (0.1%–0.2%) of SMS-biochar. This amendment significantly enhances mycelial growth rates and colonization across strains (Kittimorakul et al., 2022).

Further improvements are achieved through chemical modification. Phosphoric acid modification of SMS-biochar (AMMS) enhances its surface area, microporosity, and hydrophilicity. When this modified SMS-biochar was incorporated back into cultivation substrates for *P. ostreatus*, it functioned as a performance-enhancing additive, increasing yields by 13%–16% and reducing the harvest time by 2.5 days compared to unmodified SMS biochar, confirming an effective and safe strategy for closing the loop in mushroom cultivation (Hu S. et al., 2025). Moreover, fungi can be cultivated on elements-enriched substrates (e.g., selenium, phosphorus) to bio-accumulate them and therefore directly incorporate favorable functionalities into the resulting SMS-biochar structure for tailor-made applications. The robustness of this approach is well demonstrated by Hu et al. (2022), who established that the reintroduction of SMS-biochar into mushroom cultivation substrates significantly enhanced moisture retention, a key attribute that improved oyster mushroom yields by 20%–25% and reduced fruiting time by 4–6 days.

### 5.3 Biomedical and sensing frontiers

The biomedical use of mushroom-derived biochar made from mushrooms is gaining significance due to its tunable porosity, high surface area, and abundance of functional groups. The same properties make it a suitable drug delivery platform with controlled release-related systems for therapeutic use. Chitosan-doped biochars possess inherent antibacterial properties, and therefore, they should be utilized in tissue repair and wound healing. Biochars derived from mushrooms are also utilized as effective supports for immobilizing enzymes in biosensors, allowing for the development of sensitive diagnostic devices. Metal- and nitrogen-doped biochars derived by *in situ* growth of metal-organic frameworks (MOFs) on mushroom-derived precursors are found to be highly active for oxygen reduction reactions in Zn–air batteries and microbial fuel cells. Their hierarchical porosity and active site abundance enable them to have high power densities, stability, and enhanced ORR performance (Miao et al., 2022).

For electrochemical sensing, Liu et al. (2021) prepared a ZnO–MoO_3_–biochar nanocomposite electrode using mushroom-derived carbon nanosheets. The electrode showed sensitive and selective determination of acetaminophen in the presence of dopamine, efficient electron transfer, low detection limits, and reproducible performance in real biological and pharmaceutical samples. Similarly, nitrogen-doped biochar (N-BC) derived from mushroom bran (an SMS component) exhibited enriched surface functional groups and greater roughness, thereby enhancing its electrochemical activity. As a sensor, N-BC demonstrated high sensitivity for detecting Pb^2+^ and Cd^2+^ separately and in combination, thereby expanding the application of mushroom biochar in environmental monitoring and sensing (Yang et al., 2023).

For energy storage, architecturally designed heteroatom-doped mushroom-derived biochars show higher performance. Phosphorus- and nitrogen-co-doped cloud cap-like porous carbon derived from mushrooms was used as a cathode host material in lithium-sulfur (Li–S) batteries, exhibiting an ultrahigh surface area of 788 m^2^/g with abundant micro/mesopores that facilitate sulfur loading and polysulfide adsorption. The resulting composite exhibited an initial capacity of 1357.8 mAh/g with long-term retention of 729 mAh/g after 100 cycles at varying discharge rates and demonstrated itself to be a sustainable, high-performance conductive material (Wu et al., 2016). 800 °C pyrolyzed *L. edodes* SMS-derived- nitrogen-doped biochar was demonstrated to be a superior electrode material for zinc-ion hybrid capacitors with high specific capacitance (317.9 F/g), energy density (122.8 Wh/kg), and outstanding long-term cycling stability. This illustrates effective upcycling of low-value mushroom cultivation wastes into high-value sustainable carbon materials for future energy storage (Ni et al., 2025).

### 5.4 Carbon capture and energy applications

*Lentinula edodes* SMS-biochars derived from farm waste expand their uses beyond carbon capture and environmental restoration to agriculture and energy, demonstrating the vast range of applications for innovative functional materials. Under carbon capture, the CO_2_ adsorption capacity of SMS-biochar increased with higher pyrolysis temperatures, reflecting a greater surface area and pore volume. The incorporation of calcium at the strategic stage of biochar production also enhanced CO_2_ adsorption, indicating a feasible pathway for designing mushroom biochars for carbon sequestration (Song et al., 2024).

These carbon capture applications are secondary to energy-oriented uses. As discussed above, nitrogen-doped mushroom-derived biochars exhibit extremely high electrocatalytic activity for oxygen reduction reactions in Zn–air batteries and microbial fuel cells, highlighting their multifunctional capabilities in sustainability-based technologies (Miao et al., 2022). Mushroom-derived biochars are also significant in bioenergy generation in circular economy systems. For example, the anaerobic digestion of spent mushroom substrate (SMS) can be substantially enhanced by the addition of SMS-biochar. Compost pretreatment (CP) combined with SMS-biochar and Ce^3+^ supplementation as an integrated strategy improved AD performance by decreasing the lag period, enhancing bacterial diversity, and increasing methane production. This approach achieved a total methane yield of 69.69 L/kg VS, a 22.3% increase from the untreated SMS. The process enriches specifically targeted hydrolytic and acidogenic bacterial families (Syntrophomonadaceae, Anaerolineaceae, Ruminococcaceae), successful valorization of SMS for enhanced biogas production and energy recovery (Pan et al., 2021). Overall, the aforementioned studies show the potential of tailored mushroom biochars as multifaceted materials for carbon fixation, energy conversion, and environmentally friendly treatment of mushroom cultivation waste.

### 5.5 Current challenges and limitations

Despite the promising potential, some key challenges must be recognized and overcome for the substantial translation of laboratory-scale research into full commercial-scale application of mushroom-derived biochars. Such a critical assessment is very important for guiding future research and development.

A major challenge is the variability and standardization of feedstocks. The chemical and physical properties of SMS-biochar are highly variable, dependent on mushroom species, original substrate composition, and cultivation practices. Mycelial biochar from pure mycelium is more uniform; however, scaling up its production as a dedicated feedstock (as opposed to a waste stream) introduces economic complexity. This inherent variability poses a significant barrier to achieving the reproducible performance required in industrial applications.

The question of economic viability and scalability is closely linked to this. The economic case for mushroom-derived biochar, particularly mycelial biochar, is yet to be fully established. While using SMS represents a powerful valorization strategy for organic wastes, the collection, transportation, and processing costs of this generally bulky, high-moisture material can be infeasibly high in the absence of localized, integrated facilities. Where high-performance applications necessitate chemical activation or nano-modification, the additional processing cost has to be justified by a significant performance premium over cheaper, plant-derived alternatives. Comprehensive life cycle and techno-economic analyses are needed urgently to identify the most economically viable pathways.

The long-term environmental fate and possible ecotoxicity of these materials are also not yet well understood. While biochar itself is generally considered stable, the high nitrogen content and peculiar surface functionalities that may characterize mushroom-derived biochars could impact their degradation rate and interaction with biota in the longer term. Finally, there is some risk of ecotoxicity, especially for modified biochars such as nano-metal composites, where metal leaching or the generation of reactive oxygen species may have adverse effects on soil or aquatic microorganisms. Moreover, the effect on the soil microbiome, though often positive, is complex and has the potential to disrupt long-standing ecological balances in non-agricultural or pristine environments.

Ultimately, it is essential to recognize that performance trade-offs and application-specific limitations do exist. Properties making mushroom-derived biochar perform very well in one context may be detrimental in another. For example, the high ash and mineral content in some SMS-biochars can be advantageous for soil fertilization; however, it reduces the relatively stable carbon content, making them less ideal for dedicated carbon sequestration. Similarly, microporosity optimized for contaminant adsorption may not be the ideal architecture for microbial colonization if the pore sizes are too small. This, therefore, calls for a shift from a “one-size-fits-all” approach to the careful tailoring of biochar properties for each use case.

To overcome these limitations, standardized feedstock protocols, detailed economic modeling, long-term environmental monitoring, and application-focused design will be the next crucial steps for the field. Directly addressing these challenges, as discussed in the following “Future Outlook,” will enable the responsible and efficacious deployment of mushroom biochar technologies.

## 6 Limitations and comparative drawbacks

Despite its utility, mushroom-derived biochar has inherent limitations, particularly when compared to living fungi. The pyrolysis process destroys the complex enzymatic machinery of living mycelium, meaning biochar cannot biodegrade pollutants as white-rot fungi do; it primarily immobilizes contaminants via adsorption, leading to finite capacity and potential for secondary waste. Furthermore, biochar is a static material, lacking the self-regenerating and adaptive growth of a living fungal network that can dynamically interact with plant roots and soil ecosystems. While biochar offers rapid sorption and stability in harsh conditions, it requires significant energy for production and fails to replicate the active, enzymatic remediation and symbiotic nutrient cycling provided by living fungi. Therefore, biochar should be viewed as a complementary technology rather than a replacement, with its application chosen based on the specific requirement for a robust adsorbent versus a dynamic biological agent.

## 7 Future outlook

The future of mushroom-biochar lies in the progression from characterization to intentional, design-based engineering of mycomaterials. Future work should focus on selecting fungal strains not only for optimal food yield but for their desirable biochar properties, such as high chitin content or an innate capacity for mineral sequestration. This approach is supported by recent evidence of extensive intraspecific genetic variation. For instance, a study on the split-gill mushroom *Schizophyllum commune* demonstrated that different strains exhibit vast differences in mycelial chemical composition and material architecture, which directly translate to tunable physical properties in the derived materials (Whabi and Xu, 2025). This inherent genetic toolkit can be leveraged for the “genetic tuning” of biochar. Metabolic engineering offers the promise of designing fungi that hyper-accumulate target metals or produce specific polymers, thereby creating designer biochars pre-programmed for advanced applications in catalysis, contaminant-specific adsorption, or energy storage. Another emerging opportunity is the design of hybrid living matter, featuring biochar as a sponge-like structure for microbial communities that conduct continuous processes, such as water cleaning or soil purification. Small-scale, decentralized pyrolysis installations may also enable rural communities and mushroom farms to recycle waste on-site, providing biochar for various applications, including on-site farm use, environmental benefits, and energy production, thereby closing the loop in a true circular economy.

## 8 Conclusion

Mushroom-derived biochar is a high-value, diverse biological platform possessing applications in environmental remediation, agriculture, energy, and sensing technologies. In remediation, functionalized mushroom-derived biochars are effective at removing heavy metals, dyes, antibiotics, and emerging contaminants from soil and water. SMS-biochars and residue-derived biochars possess promise in enhancing microbial inoculation, soil fertility, crop yield, and nutrient recovery in agricultural use. In power sectors, heteroatom-doped mushroom-derived biochars are utilized as high-performance electrodes, catalysts, and supports for anaerobic digestion and bioenergy recovery. Finally, in biomedical and sensing materials, biochars provide tunable, high-surface-area materials for drug delivery, biosensing, and highly advanced electrochemical devices. By placing mushroom-derived biochar in its new context as a functional “mushroom matrix” rather than a waste material, this review highlights its potential as a keystone technology for circular economy strategies, environmental conservation, and sustainable biotechnological innovation.
